# Peripherally inserted central venous catheter in outpatient antibiotic spinal infection treatment is safe, effective and leads to significant reduction in healthcare expenses

**DOI:** 10.1007/s10143-024-03127-z

**Published:** 2024-11-23

**Authors:** Maximilian-Niklas Bonk, Björn Sommer, Philipp E. Krauß, Christoph Maurer, Valeska Simon, Ansgar Berlis, Reinhard Hofmann, Ehab Shiban

**Affiliations:** 1 Medical School, University Augsburg, Augsburg, Germany; 2 Department of Neurosurgery, University Hospital Augsburg, Augsburg, Germany; 3 Department of Neuroradiology, University Hospital Augsburg, Augsburg, Germany; 4 Department of Microbiology, University Hospital Augsburg, Augsburg, Germany; 5 Department of Neurosurgery, Lausitz University Hospital Cottbus, Cottbus, Germany

**Keywords:** Spinal infection, Spondylodiscitis, Intravenous antibiotic therapy, Outpatient, Peripherally inserted central catheter (PICC), Safety, Efficacy, Complications

## Abstract

Prolonged antibiotic therapy is often recommended for the treatment of spinal infections. This study aimed to evaluate the efficacy and safety of outpatient intravenous (IV) antibiotic therapy for spinal neurosurgery patients with spondylodiscitis. We carried out a retrospective study involving 67 patients who were administered peripherally inserted central catheter (PICC) for IV antibiotic treatment from January 2020 to December 2022. We assessed patient data concerning infections and neurosurgical concerns. Each patient underwent a minimum of 6 weeks of IV antibiotics, both as inpatients and outpatients. The study included 67 patients with a median age of 61 years (SD +/- 14.18 years), with approximately 44% being female. The average hospital stay for inpatient treatment was 20 days (SD +/- 8.8 days). Subsequent outpatient antibiotic therapy lasted an average of 70.32 days (SD +/- 18.24 days), with outpatient IV therapy accounting for 44.74 days (SD +/- 9.15 days). The most common pathogens identified were Staphylococcus epidermidis and methicillin-sensitive Staphylococcus aureus. Microbiological analysis did not detect any pathogens in 18% of patients. Radiographic and laboratory evidence of spondylodiscitis was absent in 99% of patients during the final follow-up. No catheter-related complications occurred. Outpatient IV antibiotic therapy using a PICC line catheter is a safe and effective treatment option for spinal infections, especially in elderly patients.

## Introduction

Spinal infections, such as spondylodiscitis, pose significant challenges in the field of neurosurgery [1–3]. Prolonged antibiotic therapy is commonly recommended for effective management. In recent years, there has been a growing interest in the outpatient administration of intravenous antibiotics, aiming to reduce hospital stay durations and associated costs. Notably, in Germany, early evidence of this practice emerged in 2020 when researchers from Jena published their findings on administering chemotherapy to 512 patients via a PICC line [4]. This study’s goal was to evaluate the efficacy and safety of outpatient intravenous antibiotic therapy in spinal neurosurgery patients with spondylodiscitis.

This gap in knowledge has motivated our team to rigorously evaluate and scientifically document our experiences in this domain, with the aim of contributing valuable insights to the field.

## Methods

We conducted a retrospective single-center study with peripherally inserted central catheter (PICC) for IV antibiotic therapy between January 2020 and December 2022 at the Department of Neurosurgery. Patient data related to infectious and neurosurgical issues were evaluated. Based on the findings from the “Duration of Treatment for Spondylodiscitis (DTS) study group” and the publications by Bernard et al., the protocol for our study involved administering intravenous antibiotics to all patients for a minimum duration of six weeks [2, 3, 5–7]. This treatment regimen was applied to both inpatients and outpatients, aligning with the DTS study group’s recommendations and findings. This approach was informed by their extensive research and conclusions on the optimal duration of antibiotic therapy for effectively managing spondylodiscitis [5]. We used the STROBE statement as a reporting guideline [8].

### Patients

The study focused on patients who underwent peripherally inserted central catheter (PICC) insertion for intravenous antibiotic therapy in the treatment of spinal infections. A total of 67 patients were included in the study, with no instances of lost to follow-up. All patients attended regular follow-up appointments.

In this study, the insertion of the PICC lines was not only performed by experienced (neuro-)radiologists under sterile conditions but also scheduled promptly to ensure continuous intravenous antibiotic therapy. Specifically, all patients had the catheter inserted into the brachial vein of their non-dominant arm. This early placement was crucial to maintain uninterrupted treatment. Furthermore, each patient provided written consent before the PICC line insertion, ensuring that all procedures were conducted with informed patient agreement, adhering to ethical medical practices. This careful approach reinforced the study’s commitment to patient safety and ethical standards.

The study encompassed all patients treated for spinal infections, regardless of whether they underwent surgery or received conservative management. After discharge, patients were closely monitored through a structured outpatient care program. This included weekly monitoring of inflammation markers by their primary care physicians and regular PICC line maintenance by nursing services. The PICC line dressing (holding plate) was changed on a weekly basis by the nursing staff, or more frequently in cases where contamination or soiling occurred.

Additionally, primary care physicians were responsible for prescribing the necessary antibiotics. At the 6-week mark, patients were scheduled for a follow-up appointment in our clinic, where they underwent updated MRI and CT scans. Based on these evaluations, decisions regarding the transition from intravenous to oral antibiotic therapy were made. This comprehensive approach ensured continuous and thorough patient care post-discharge.

The study established specific inclusion and exclusion criteria for patient selection:

**Table:** 

Inclusion Criteria	Exclusion Criteria
Patients aged over 18 years	Patients requiring internal transfer to another department for further treatment (e.g., cardiothoracic surgery)
Capability to provide informed consent	A positive history of drug use
Assurance of reliable outpatient care	

**Fig. 1:**
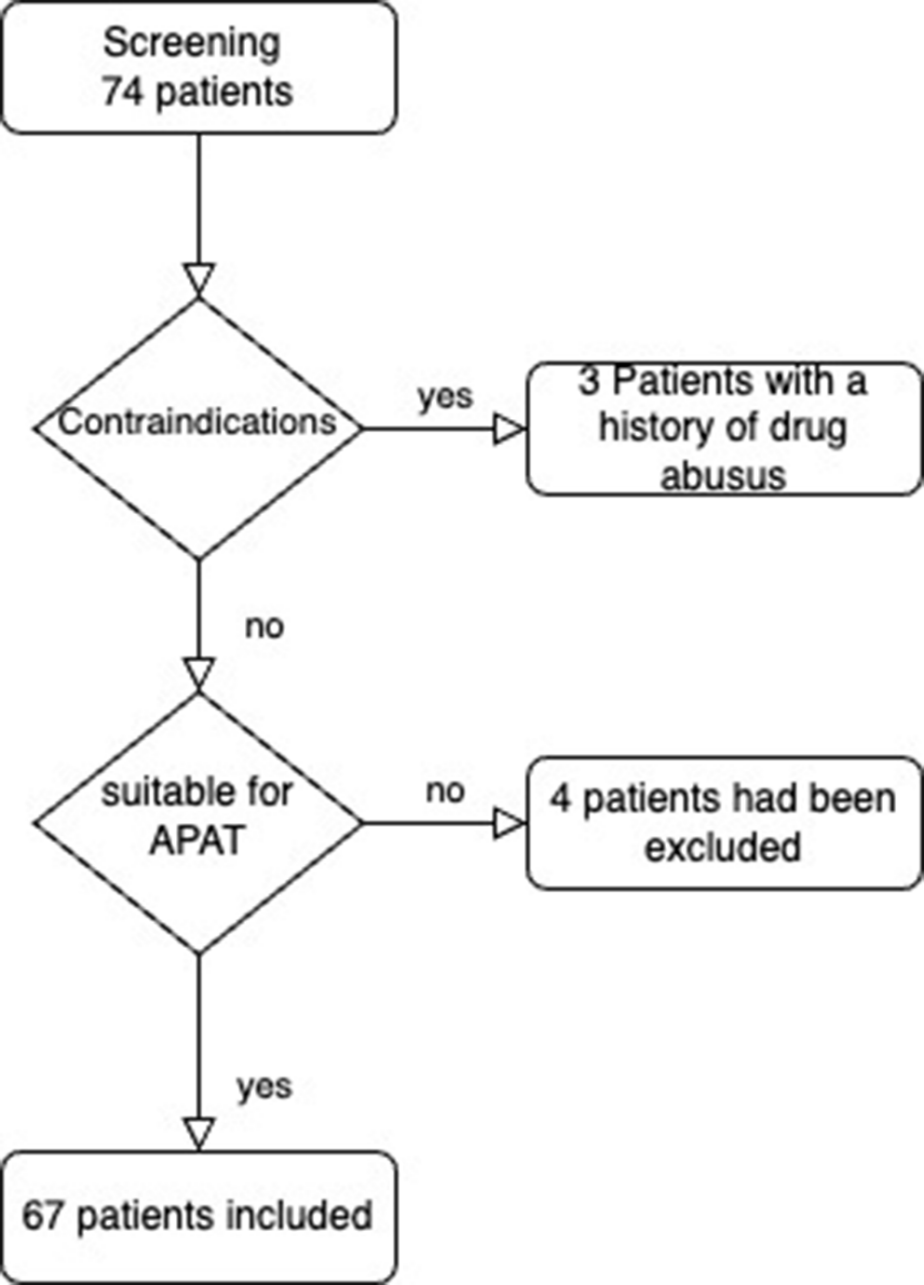
Flowchart patient recruitment

During the initial screening of 74 patients, 7 were excluded based on these criteria. Specifically, three patients were excluded due to a positive drug history. Two patients required internal transfer to other specialized departments for additional treatments. For two other patients, a secure outpatient care setup could not be ensured. This careful selection process was crucial to maintain the integrity and focus of the study (Fig. 1).

### Data collection

Data collection was performed using our digital hospital information system (ORBIS, Dedalus HealthCare Konrad-Zuse-Platz 1–3, 53227 Bonn) and the PACS system (DeepUnity, Dedalus HealthCare Konrad-Zuse-Platz 1–3, 53227 Bonn). Patient medical records encompassed a range of information such as clinical notes, operation theatre records, and ICD codes. Additionally, demographic data, including age, sex, co-morbidities, duration of symptoms, symptoms, previous therapy (medical/surgical), laboratory results, and radiological imaging (CT, MRI), were extracted. Hospital stay details, such as antibiotic drug treatment, blood results, adverse events, complications, mobility, and treatment success, were also documented. Follow-up data included physical examinations, blood tests, MRI, and CT scans, along with medical documentation.

### Treatment approach

All treatment decisions in this study were made in accordance with current guidelines for the management of spinal infections. Regardless of whether patients underwent surgery or not, the overall treatment protocol remained consistent across all cases. This included the administration of appropriate antibiotics, pain management, and immobilization as needed.

Pain management was a crucial aspect of patient care throughout the treatment process. All patients received adequate analgesic therapy, which was carefully titrated to ensure optimal pain control before discharge. At the time of discharge, patients had achieved good pain compensation through oral medications, including extended-release formulations where appropriate. During outpatient follow-up visits, the co-medication regimen, including analgesics, was critically reviewed alongside the antibiotic therapy. Patient satisfaction with pain management was consistently assessed during these visits. Furthermore, we maintained close communication with each patient’s primary care physician, who had the authority to optimize pain management if necessary. This comprehensive approach to pain control ensured that patients received continuous and effective pain relief throughout their treatment and recovery period, both as inpatients and during outpatient care.

The post-treatment care and follow-up were identical for both surgical and non-surgical groups, involving regular clinical assessments, laboratory tests, and imaging studies. The primary difference in treatment was the surgical intervention itself; all other aspects of care, including the duration and type of antibiotic therapy, outpatient management, and long-term follow-up, were standardized across the entire cohort.

All treatment decisions in this study were based on the Spinal Infection Severity Score (SISS), ensuring a standardized approach for both conservative and surgical cases [9]. Patients who were managed conservatively were those who did not meet the criteria for surgical intervention, as determined by their SISS classification. Typically, surgical management was reserved for more severe cases with higher scores indicating the need for operative treatment.

A key component of the treatment process was physiotherapy, which was provided daily from Monday to Friday during the hospital stay. Post-discharge, patients were given recommendations to continue physiotherapy in an outpatient setting to support their recovery. This consistent integration of physiotherapy aimed to enhance mobility, reduce pain, and support overall recovery, regardless of the treatment method applied.

The duration of both the initial Ambulatory Parenteral Antibiotic Therapy (APAT) and the subsequent oral antibiotic therapy was determined based on the recommendations provided by the microbiology department. This tailored approach ensured that the most appropriate treatment regimen was applied to each individual patient.

Monitoring was conducted regularly using inflammatory markers (such as C-reactive protein, white blood cell count, and erythrocyte sedimentation rate) and magnetic resonance imaging (MRI) at the conclusion of APAT or at the planned end of oral antibiotic therapy. If any abnormalities or signs of persistent infection were detected, the duration of the antibiotic therapy was extended accordingly. This approach allowed for adaptive treatment durations, ensuring optimal patient outcomes while minimizing the risk of relapse.

### Surgical approach

The surgical approach for each patient was determined on an individual basis, taking into account several key factors, including comorbidities, the location of the infection, the presence or absence of epidural spinal cord compression, neurological compromise, and the extent of bony destruction. In cases where the endplates were intact but there was reduced disc height, the primary concern was the potential for spinal instability. This necessitated careful consideration of the surgical approach.

For patients with signs of instability, we had to weigh the need for a more extensive 360° fusion versus a less invasive posterior dorsal instrumentation. In many cases, posterior pedicle screw instrumentation with decompression was performed when there was sufficient stability, as indicated by the patients’ SISS score. However, when spinal deformity or extensive vertebral body destruction was present, a more invasive approach, such as an extreme lateral or anterior transperitoneal approach, was selected for vertebral body replacement and fusion to provide greater stability.

Pure decompression was reserved only for cases involving isolated spinal abscesses, where no instability was evident, and the SISS score justified a less invasive approach. Overall, the decision-making process balanced the need for stability with the extent of the surgical intervention required to ensure optimal patient outcomes.

### Microbiological analysis and antibiotic therapy

#### Microbiological sampling and processing

All microbiological samples were processed and analysed according to standardized protocols at the Institute for Microbiology at the University Hospital Augsburg. To ensure the accuracy of results and minimize the risk of contamination, a rigorous approach was implemented:

1. Each positive culture was critically evaluated for plausibility by both the microbiology team and in joint discussions between microbiologists and neurosurgeons.
2. A strict requirement for double independent pathogen detection was enforced. This could include:

- Positive blood culture and/or surgical site swab.
- Multiple detections of the same pathogen from different patient sites (e.g., urine, bronchoalveolar lavage).
- Independent samples (e.g., blood culture pairs taken at different time points).

This approach was particularly crucial in cases where potentially contaminant organisms, such as Staphylococcus epidermidis, were isolated, ensuring that these represented true infections rather than sample contamination.

#### Focal search protocol

In addition to microbiological sampling, a standardized infection search protocol was followed to identify the source of infection. This multi-step approach ensured thorough diagnostics and minimized the risk of missed infections. The steps were as follows:

Initial diagnostics:

- Multiple blood cultures were taken from various anatomical sites.
- Imaging studies, including CT scans of the head, sinuses, and teeth, were performed. Based on these results, referrals to ENT, maxillofacial surgery, or dental specialists were made if necessary.
- Further diagnostic tools included abdominal ultrasound and chest X-ray, alongside comprehensive infection serology (HIV, Hepatitis A/B/C, syphilis). Additionally, transesophageal echocardiography (TEE) was performed to exclude endocarditis as a source of infection.

Foreign material evaluation:

For patients with implanted devices such as ports or joint prostheses (hip/knee), explantation was performed, and the material was sent for microbiological analysis. This step was undertaken in consultation with relevant specialties to ensure proper handling and analysis of the explanted devices.

Advanced diagnostics:

If no source of infection was identified through the initial steps, or if there was suspicion of an ongoing infection despite negative findings, we consulted with the department of nuclear medicine. Advanced imaging techniques such as FDG-PET CT or leukocyte scintigraphy were employed to locate the source of infection.

This third step was only performed when the previous steps failed to identify the infection focus, ensuring that invasive or complex diagnostics were used sparingly and only when necessary.

#### Antibiotic selection and management

Antibiotic selection and dosing regimens were carefully coordinated with the microbiology department to ensure effective therapy while considering the practicalities of outpatient administration. The antibiotics were chosen based on the recommendations of European Association of Neurosurgical Societies (EANS) with a particular emphasis on the use of narrow-spectrum antibiotics to minimize the risk of side effects [10].

While the aim was to limit dosing frequency for convenience in the outpatient setting, the best possible antibiotic choice was prioritized for each case, even if this required multiple daily doses.

Key aspects of the antibiotic management included:

1. Emphasis on narrow-spectrum antibiotics to minimize the risk of side effects and antibiotic resistance.
2. Careful adjustment of antibiotic dosing before patient discharge to ensure optimal efficacy.
3. Blood level monitoring for certain antibiotics, such as Gentamycin and Vancomycin, to maintain safe and effective concentrations.
4. Development of a solid and well-monitored treatment plan for each patient upon discharge.

### Primary endpoint

The primary endpoint of the study was the rate of healing or full recovery, which was defined as:

1. No radiographic evidence of persistent infection observed in MRI scans of the spinal column.
2. No elevation in white blood cell count (WBC), C-reactive protein (CRP), alkaline phosphatase, and erythrocyte sedimentation rate.
3. Marked reduction in pain.

### Secondary endpoints

1. Evaluation of morbidity and mortality rates related to the disease, surgical interventions, or hospitalization.
2. Analysis of complications arising from outpatient antibiotic therapy.
3. Assessment of health-related quality of life (HRQoL) and overall survival (OS) in patients receiving ambulatory parenteral antibiotic therapy.
4. Examination of the feasibility of this therapy for outpatient patients, especially older individuals.

These additional secondary endpoints will provide a comprehensive analysis of both clinical outcomes and the broader impact of the treatment on patients’ quality of life and overall well-being.

### Ethical considerations

The study protocol was approved by the ethic committee of the Ludwig-Maximilian-University Munich (Bavaria / Germany) on May 26th, 2023, reference number 2023 − 0304.

### Statistical analysis

Descriptive statistics, inferential tests (e.g., t-tests, chi-square tests), and regression analyses were conducted as appropriate based on the nature of the variables and research questions. Statistical software (GraphPad PRISM 9; GraphPad Software Boston (MA) USA) was employed for data analysis, and the significance level was set at *p* < 0.05. Values were reported using median and one standard deviation (±1SD). We checked Gaussians distribution using the Shapiro-Wilk test. Statistical differences of metric variables (blood loss, duration of preoperative symptoms, length of hospital stay) were calculated using either the Student´s t-test or the Wilcoxon test for paired samples and the Mann-Whitney-U test for unpaired samples.

Distribution of categorial variables (vertebral body subsidence (yes/no), fusion rate (yes/no), healing rate (yes/no)) in our samples were calculated using the chi squared test with contingency tables.

## Results

A total of 67 patients (26 female, 41 male, age 61±14.2 years) were included. The average length of hospital stay for inpatient treatment was 20 ±8.8 days. After discharge from hospital, the patients underwent outpatient antibiotic therapy for an average duration of 70.32 ±18.24 days. Out of this duration, intravenous (IV) therapy was administered for an average of 44.74 ±9.15 days).

**Fig. 2:**
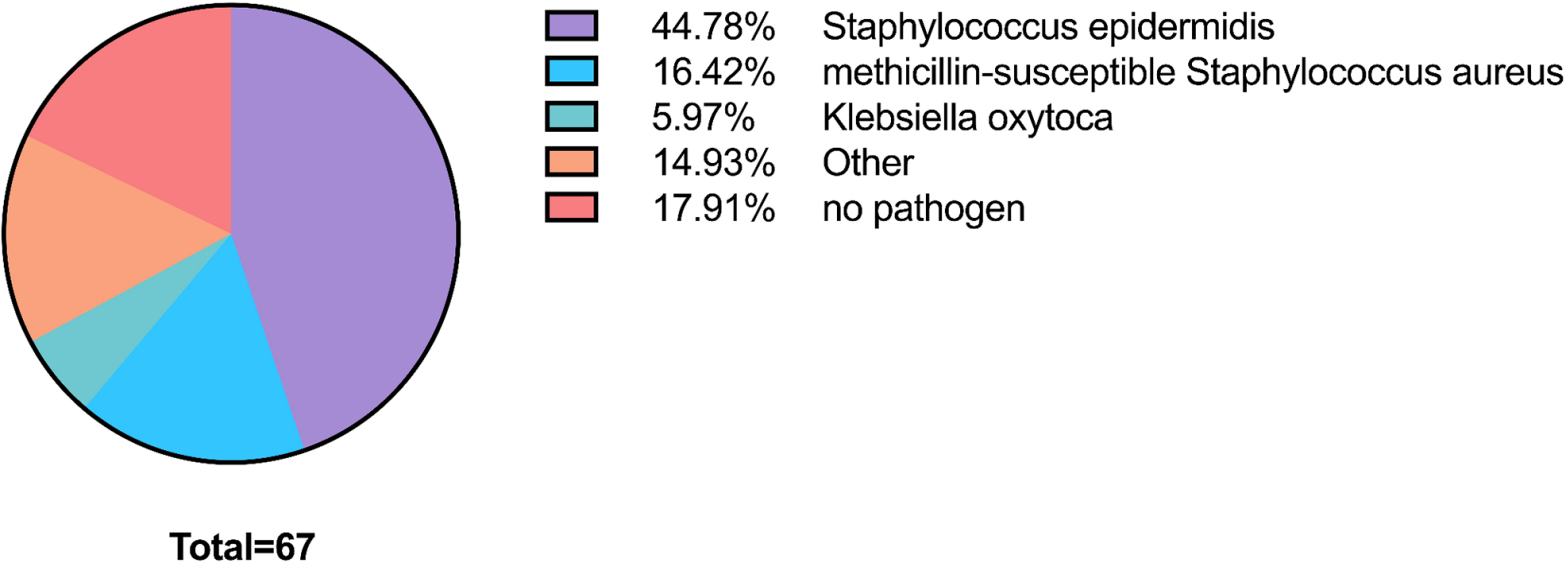
Overview of pathogens

The most frequently identified pathogens were *Staphylococcus epidermidis* and methicillin-sensitive *Staphylococcus aureus*. However, it is noteworthy that in 18% of the patients, no pathogens were detected through microbiological analysis. During the final follow-up, radiographic and laboratory chemical evidence of spondylodiscitis was absent in 99% of the patients. *Staphylococcus epidermidis* was consistently found in blood cultures and never in material from explanted devices, and after thorough investigation, we confirmed these cases as true infections (Fig. 2).

None of the patients in the study required a replacement of their PICC line during the course of treatment. Additionally, none of the catheters reached the manufacturer’s recommended maximum lifetime of 3 months, as all patients completed their antibiotic therapy before this limit was reached.

### Distribution of spinal infections

Magnetic resonance imaging (MRI) confirmed spondylodiscitis in all 67 patients included in the study. The distribution of infection sites revealed interesting patterns. In 65.7% (*n* = 44) of cases, the infection was confined to the intervertebral disc and adjacent osseous structures, representing classic spondylodiscitis. The remaining 34.3% (*n* = 23) of cases demonstrated a more extensive infection pattern, with unilateral or bilateral involvement of the psoas muscle, indicating paraspinal spread. Notably, intraspinal abscess formation, a severe complication of spinal infections, was observed in only 8.7% (*n* = 6) of cases.

The distribution of spinal infection cases across regions of the spine was as follows:

- Cervical spine: 5 cases.
- Thoracic spine: 8 cases.
- Lumbar spine: 54 cases.

However, there was one case of a patient who experienced a complication associated with the catheter during outpatient IV therapy. This patient developed a soft tissue abscess. This case was managed appropriately, with no further complications arising from the catheter usage.

Importantly, none of the patients required revision surgeries as part of the overall treatment. Additionally, there were no instances of implant-related issues, such as screw malposition or implant failure, nor were there any cases of wound healing disturbances or other surgery-associated complications.

**Fig. 3:**
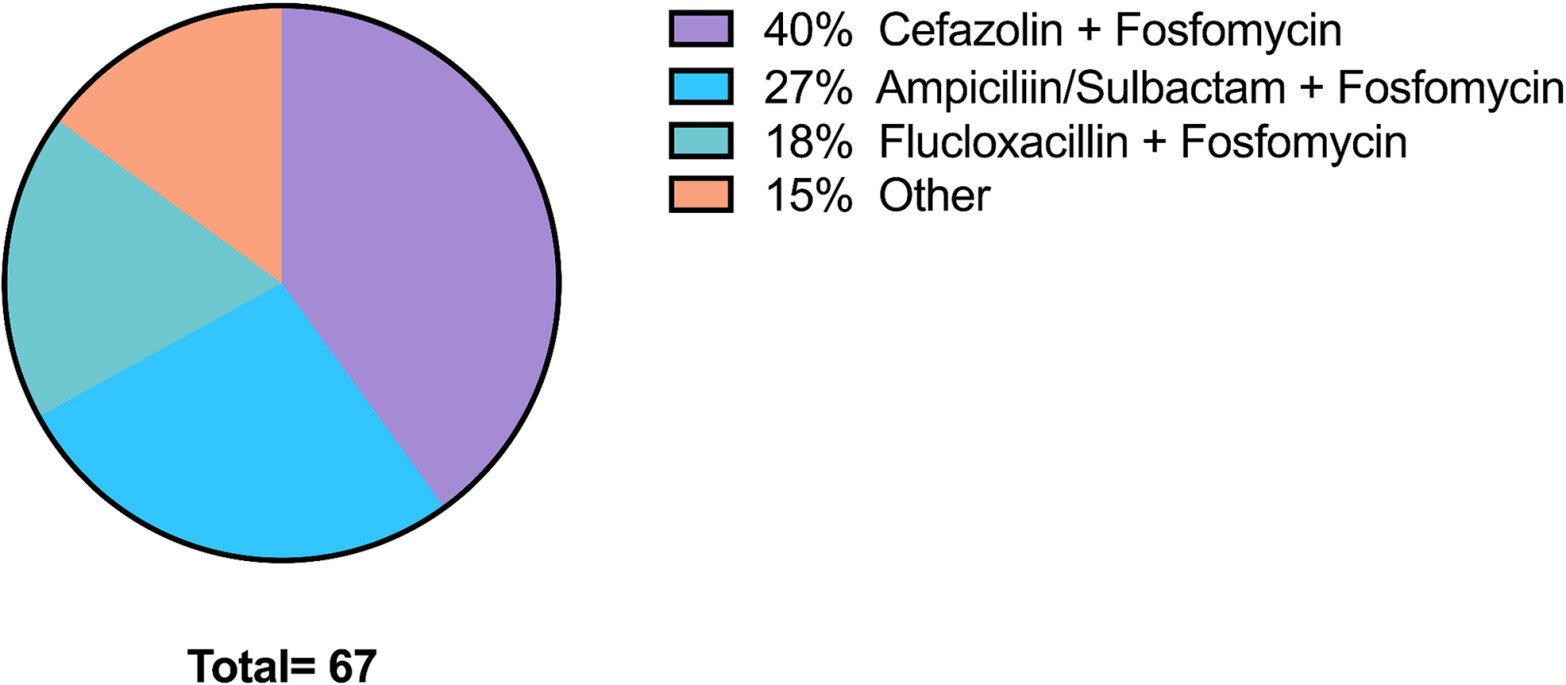
Distribution of used antibiotics

Patient and caregiver feedback indicated that even multi-dose regimens were manageable, with positive reports regarding the practical aspects of administration. A breakdown of the most frequently used antibiotics showed that Cefazolin combined with Fosfomycin was the most commonly administered combination, followed by Ampicillin/Sulbactam with Fosfomycin and Flucloxacillin with Fosfomycin, as illustrated in the following diagram (Fig. 3).

Notably, none of the patients experienced any adverse drug reactions directly attributable to the antibiotics used in their treatment regimens. Furthermore, there were no instances of indirect side effects commonly associated with antibiotic use, such as elevated retention parameters or increased liver enzymes.

### Neuological status

At the time of admission, 64 patients were classified as ASIA D or E, indicating that they were ambulatory. Importantly, there were no cases of neurological deterioration during the course of treatment, and all 64 patients remained ambulatory at the time of discharge.

Additionally, 3 patients presented with incomplete paraplegia, classified as ASIA C. Of these, 2 patients showed significant improvement, recovering to ASIA E, while 1 patient improved to ASIA D by the time of discharge.

### Surgical interventions

Of the 67 patients included in the study, 48 (71.7%) underwent primary dorsal instrumentation as part of their treatment for spinal infection. The distribution of surgical interventions across the spine regions was as follows:

- Cervical spine: 4 patients.
- Thoracic spine: 4 patients.
- Lumbar spine: 40 patients.

Among these surgically treated patients, further interventions were necessary in some cases. Specifically, 17 patients (36.36% of those who had primary dorsal instrumentation, or 25.37% of the total cohort) required an additional lateral approach procedure. Furthermore, 4 patients (9.09% of those with primary dorsal instrumentation, or 5.97% of the total cohort) needed vertebral body replacement. These figures highlight the complexity of surgical management in spinal infections, demonstrating that while dorsal instrumentation was the primary surgical approach, a significant proportion of patients required additional surgical interventions to effectively manage their condition. The remaining 19 patients (28.3% of the total cohort) were managed conservatively without surgical intervention.

### Comparative analysis of surgical and non-surgical groups

A detailed statistical analysis was conducted to compare the outcomes between patients who underwent surgical intervention and those who were managed conservatively. Of the 67 patients, 48 (71.7%) received surgical treatment, while 19 (28.3%) were treated non-surgically. The analysis focused on both direct and indirect parameters during the inpatient stay and throughout outpatient care. Direct parameters included infection resolution rates, pain levels, and functional recovery, while indirect parameters encompassed length of hospital stay, duration of antibiotic therapy, and incidence of complications.

The comparative analysis revealed no significant differences between the surgical and non-surgical groups regarding these parameters. Both groups demonstrated similar outcomes in terms of infection control, pain management, and overall recovery.

### Financial savings and reduced hospital stays

#### Financial implications

Our financial analysis, conducted in collaboration with the hospital’s financial control department, focused specifically on calculating the cost savings achieved by reducing inpatient stays through our outpatient antibiotic therapy (APAT) intervention. The estimated savings amounting to approximately €2.7 million represent the direct financial benefit to the hospital, derived from the costs that would have otherwise been incurred if patients had remained hospitalized for the entire course of antibiotic treatment.

#### Calculation of hospital cost savings

The savings calculation was based on the following methodology:

- **Calculation of hospital days saved**: For each patient, we determined the number of hospital days saved by subtracting the actual length of inpatient stay from the total duration of the prescribed antibiotic therapy.
- **Estimation of daily inpatient costs**: The hospital’s average daily inpatient care costs were applied to estimate the expense saved per day. These costs included nursing, room occupancy, and general inpatient resources, excluding the cost of antibiotics, as these would be incurred whether the therapy was administered inpatient or outpatient.
- **Total savings calculation**: The number of saved hospital days was multiplied by the daily inpatient cost to determine the total hospital savings.

The resulting figure of €2.7 million reflects purely the cost savings from hospital resources freed up by transitioning suitable patients to outpatient antibiotic therapy. This calculation deducts the expenses related to the PICC line catheter and the physician’s time required for outpatient care coordination, providing a net savings estimate for the hospital alone. to approximately €2.7 million.

#### Limitations of the cost analysis

It is essential to clarify that this calculation focuses solely on the hospital’s cost savings, excluding costs and potential savings related to the broader healthcare system. For instance, while hospital costs were reduced, the shift to outpatient care introduces other expenses that are more challenging to quantify comprehensively. These include:

- **Outpatient clinic visits**: Patients required regular follow-up visits, typically managed by general practitioners or outpatient specialists, to monitor their response to therapy and assess their PICC lines. These visits entail additional costs borne by the healthcare system.
- **Primary care workload**: General practitioners often manage follow-up care in outpatient settings, adding to their workload. This extra demand on primary care resources could entail indirect costs for healthcare providers outside the hospital.
- **Patient transportation and time costs**: Transitioning to outpatient care means patients are responsible for their own transportation to follow-up visits, which can be costly, especially for those living far from healthcare facilities. Additionally, patient-borne costs like time off work are not factored into our hospital-centric calculation.
- **Outpatient laboratory costs**: Patients typically required periodic lab tests to monitor their response to therapy. These tests, performed at outpatient labs or primary care facilities, introduce costs outside the hospital’s purview.

Given the complex variability in outpatient expenses, particularly regarding patients’ insurance status, travel distances, and individual care needs, estimating the complete cost impact on the healthcare system was outside the scope of our analysis. Our primary focus was to quantify the financial savings specifically realized by the hospital.

In summary, while the €2.7 million in savings reflects a significant reduction in hospital costs due to APAT, it does not necessarily translate into equivalent savings for the broader healthcare system.

#### Increased hospital capacity

The reduction in inpatient days also translated into increased hospital capacity. This additional capacity allowed for the admission of new patients, potentially improving overall healthcare system efficiency. However, quantifying the exact impact of this increased capacity on the broader healthcare system remains challenging and was beyond the scope of this study.

In summary, while our intervention resulted in significant cost savings for the hospital in terms of reduced inpatient care days, it’s important to view these savings within the context of overall healthcare resource utilization.

## Discussion

Our study’s primary contribution lies in demonstrating that APAT, combined with tailored follow-up, is not only feasible but effective in both surgical and non-surgical cases of spinal infection. A recent meta-analysis from the EANS Spine Section underscored the benefits of early surgical intervention in operable cases, suggesting that early surgery leads to superior outcomes compared to extended conservative therapy alone [11]. This recommendation has emphasized the importance of distinguishing cases that would benefit from surgical versus conservative management. However, our study addresses a crucial gap in the literature by focusing on the post-treatment phase, where APAT can be integrated as a supportive or alternative approach in managing infections beyond the initial treatment decision.

In contrast to previous studies, which primarily focus on the choice between surgery and conservative management, our study shifts the emphasis to the role of antibiotic management and its effectiveness in the outpatient setting. By showing that APAT, when guided by inflammatory markers and MRI monitoring, is a safe and viable adjunct to inpatient care, we offer a practical framework for spinal infection management that can be applied in real-world clinical settings. For the first time, we present evidence that structured post-treatment APAT can be successfully employed, even in cases requiring surgery, thereby providing a new perspective on managing operable and non-operable spinal infections.

### Advantages and challenges of outpatient IV therapy

While APAT offers clear benefits, its success relies on meticulous monitoring and comprehensive patient education regarding PICC line management. Risks associated with PICC lines—such as catheter-related infections and thrombosis—require patients and caregivers to be well-informed about catheter maintenance, infection signs, and appropriate responses to adverse events. Previous studies indicate a global PICC line complication rate of 30.2%, with a higher incidence in inpatients compared to outpatients, and an overall infection rate of 2.3 per 1,000 PICC days [12]. Our findings emphasize that proactive education and consistent follow-up are critical in minimizing these risks, ultimately enhancing the safety and effectiveness of outpatient IV antibiotic therapy. This aligns with research by Liscynesky et al. (2017) and the KTFIXPICC study, both of which affirm the safety and practicality of PICC lines in both inpatient and outpatient settings [13, 14].

Additionally, studies by Lam et al. (2018) and Mielke et al. demonstrate the importance of identifying predictors for PICC line occlusion and highlight the utility of PICC lines in outpatient settings for various medical needs, such as chemotherapy [4, 15]. Our study’s results align with these findings and underscore that with adequate monitoring and education, APAT can be a secure and effective modality for treating spinal infections.

### Financial implications

One of the most compelling aspects of our study is the economic impact of APAT, which was highlighted through collaboration with our hospital’s financial control department. By reducing the duration of inpatient stays, APAT generated an estimated savings of €2.7 million, reflecting a significant financial advantage for the hospital. These savings stem from a reduction in inpatient costs, primarily due to optimized nursing resources, fewer required infusions, and minimized need for dedicated hospital facilities for prolonged care.

This economic benefit aligns with a broader trend in healthcare towards more cost-effective treatment models. For example, the study by Vargas-Palacios et al. in Leeds demonstrated substantial cost savings with outpatient IV therapy, showing that comparable levels of care quality could be maintained with significantly lower financial outlay [16]. Similarly, Staples’ research in Vancouver provides insights into the financial and logistical benefits of outpatient treatments, underscoring that these approaches can alleviate resource constraints within hospital systems [17]. Our study adds valuable data to these findings by demonstrating that APAT, with appropriate follow-up care, is not only effective but also a financially viable approach to managing spinal infections.

### Strategic healthcare management

The broader implications of our findings extend to strategic healthcare management. By reducing inpatient days, APAT can increase hospital capacity, allowing facilities to admit and treat more patients without expanding physical resources. This is especially relevant in healthcare systems facing challenges related to hospital bed availability and resource allocation. By optimizing nursing staff utilization and minimizing indirect costs associated with prolonged hospital stays, APAT can be a strategic component of patient-centered care models, particularly in specialized settings such as neurosurgery and infectious disease management.

The flexibility that APAT provides enables hospitals to address bed shortages while delivering high-quality, patient-focused care. Our study underscores that APAT is not only beneficial for individual patient outcomes but also serves as a model for more efficient healthcare management, offering a sustainable approach to managing complex infections in both surgical and non-surgical patients.

### Future directions

While our study provides promising evidence in support of APAT, it also highlights the need for further research to validate and extend these findings. Larger, prospective studies with longer follow-up periods are essential to confirm the long-term safety and efficacy of APAT for spinal infections. Additionally, future research should examine patient satisfaction, quality of life outcomes, and the broader economic impacts of APAT on healthcare systems. This would provide a more comprehensive understanding of APAT’s potential to improve patient outcomes and healthcare sustainability.

In conclusion, our study supports APAT as a viable, cost-effective alternative to inpatient care for spinal infections, emphasizing the importance of patient education, vigilant monitoring, and strategic healthcare management. By focusing on these areas, APAT can enhance treatment efficacy while ensuring patient safety and satisfaction, thus offering a new and impactful approach to managing spinal infections effectively in both surgical and conservative cases.

## Conclusion

In conclusion, this study demonstrates that outpatient IV antibiotic therapy utilizing a PICC line catheter is a safe and effective treatment option for spinal infections, particularly in elderly patients. The use of a PICC line catheter allows for a reduction in hospital stay duration while maintaining satisfactory treatment outcomes. However, close monitoring and proper education regarding the use of the catheter system are essential to prevent potential complications. Further research is needed to validate these findings and assess long-term outcomes, as well as to explore the economic and patient satisfaction aspects of this treatment approach.

## Data Availability

No datasets were generated or analysed during the current study.
